# Movement-Based Mindfulness vs. Attention Control for Modifying Physiological Risk in Chronic Stroke: Evidence from a Feasibility Trial

**DOI:** 10.3390/healthcare13222940

**Published:** 2025-11-17

**Authors:** Tharshanah Thayabaranathan, Marina Paul, Frederick R. Walker, Shaun Hancock, Liam Allan, Maarten A. Immink, Susan Hillier, Monique F. Kilkenny, Amy Brodtmann, Emma Gee, Leeanne M. Carey, Rene Stolwyk, Julie Bernhardt, Michael Nilsson, Dominique A. Cadilhac

**Affiliations:** 1 School of Clinical Sciences at Monash Health, Monash University, Clayton, VIC 3168, Australiadominique.cadilhac@monash.edu (D.A.C.); 2 Centre of Research Excellence in Stroke Rehabilitation, Heidelberg, VIC 3084, Australia; 3 Centre of Research Excellence in Aphasia Recovery and Rehabilitation, Melbourne, VIC 3086, Australia; 4 Heart and Stroke Research Program, Hunter Medical Research Institute, New Lambton Heights, NSW 2305, Australia; 5 College of Health, Medicine and Wellbeing, The University of Newcastle, Newcastle, NSW 2308, Australia; 6 Centre for Rehab Innovations, University of Newcastle, Callaghan, NSW 2308, Australia; 7 Australian e-Health Research Centre, Commonwealth Scientific and Industrial Research Organization, Brisbane, QLD 4006, Australia; 8 College of Nursing and Health Sciences, Flinders University, Adelaide, SA 5000, Australia; 9 Innovation IMPlementation and Clinical Translation in Health (IIMPACT), Allied Health and Human Performance, University of South Australia, Adelaide, SA 5000, Australia; 10 Stroke and Critical Care theme, the Florey Institute of Neuroscience and Mental Health, University of Melbourne, Heidelberg, VIC 3084, Australia; 11 School of Translational Medicine, Monash University, Melbourne, VIC 3168, Australia; 12 Survivor of Stroke, Inspirational and Motivational Speaker, Cotham, VIC 3101, Australia; 13 School of Allied Health, Human Service and Sport, LaTrobe University, Melbourne, VIC 3086, Australia; 14 School of Psychological Sciences, Monash University, Clayton, VIC 3800, Australia

**Keywords:** mindfulness, stroke, feasibility trial, biomarkers, risk factors, blood pressure

## Abstract

**Background**: Managing physiological risk factors (blood pressure, lipids, stress, blood glucose) post-stroke is essential yet challenging. In this sub-study of a feasibility randomized controlled trial, we examined changes in these parameters following a stroke-tailored mindfulness-based intervention (MBI) compared with an attention control. **Methods**: Participants 3–18 months post-stroke were recruited from the Australian Stroke Clinical Registry (May 2021–July 2023) and randomized 1:1 to a 12-week MBI (weekly yoga + ≥3 home meditation sessions) or attention control (education and peer support). All attended weekly 60-min classes. Outcomes included blood pressure and stress (perceived stress scale, hair cortisol). A sub-group (n = 17) also had HbA1c and lipid profile assessed. Descriptive statistics, within-group effect sizes (Cohen’s d), and generalized linear modeling were used. **Results**: A total of 38 participants were randomized with 36 participants completing the trial (mean age 69 years; 72% male), and the MBI group showed greater within-group improvements in blood pressure, with clinically meaningful reductions in systolic (5 mmHg; d = 0.35) and diastolic (4 mmHg; d = 0.41) values, compared to smaller effects in the control group. Exploratory trends suggested favorable change in lipid profiles (HDL and LDL) in the MBI group, while triglycerides improved in the control group. No changes were observed for HbA1c. Stress markers, including hair cortisol, showed positive trends in the MBI group (d = 0.52). No significant between-group differences were detected. **Conclusions**: This sub-study of a well-designed, rigorous feasibility trial provides preliminary findings of clinically meaningful differences in blood pressure and lipid profiles in the MBI group. The findings support the potential of MBIs in managing post-stroke cardiovascular risk factors and warrant larger trials to confirm these preliminary effects.

## 1. Introduction

Stroke is a leading cause of long-term disability and mortality in Australia and globally, with survivors at high risk of recurrence, estimated at 11% within one year and 26% within 5 years [1,2]. A second stroke often results in more severe disability or death, underscoring the need for effective prevention [3]. Survivors often struggle to manage physiological risk factors such as elevated blood pressure, blood lipids, and blood glucose, and stress, which increase recurrence risk [4]. Effective management is crucial for reducing subsequent events and improving post-stroke quality of life [4,5]. Traditional approaches to post-stroke support to manage risk factors have primarily focused on pharmacological interventions and lifestyle modifications. However, there is a growing interest in complementary therapies that address physical, physiological, and psychological health [6,7]. Despite these advances, a significant gap exists in long-term, community-based support for people living with stroke, and only a few therapies have been developed specifically for people with stroke.

Movement-based mindfulness interventions (MBIs), such as yoga and tai chi, have gained attention for their potential benefits in managing cardiovascular and metabolic health in people with chronic illnesses, including stroke [7,8,9]. Practices within these interventions emphasize low-impact physical movements, breath regulation, and focused attention, promoting relaxation and enhancing autonomic regulation [10]. Emerging evidence suggests that MBIs may moderate physiological parameters, such as reducing systolic blood pressure, cortisol levels, and blood glucose, through parasympathetic activation and decreased sympathetic arousal [7,11,12]. Additionally, MBIs have been associated with improved lipid profiles, including reductions in total cholesterol (TC) and low-density lipoprotein (LDL) levels, and increases in high-density lipoprotein (HDL) [7,13,14]. Given the elevated cardiovascular risk in people with stroke, MBIs may be a valuable addition to current medical approaches to risk reduction strategies.

Another important factor influencing physiological responses is chronic stress, known to cause inflammation in the body, which in turn exacerbates various diseases, including hypertension, dyslipidemia, obesity, and depression, thereby increasing the risk of adverse cardiovascular events [15]. There is systematic review-level evidence that MBIs may be effective in reducing perceived stress and psychological symptoms such as anxiety and depression among survivors of stroke [16,17]. These programs also contribute to physiological benefits by lowering stress hormones like cortisol, which regulates blood pressure and glucose metabolism [18]. Biological mechanisms linking MBIs to physiological outcomes include regulation of the hypothalamic-pituitary-adrenal axis and autonomic balance, both of which influence blood pressure, lipid metabolism, and glucose control through reductions in sympathetic activation and inflammatory pathways [19,20,21]. While the psychological benefits of MBIs in survivors of stroke are increasingly recognized (e.g., reduced anxiety, depression, stress), their direct physiological effects remain underexplored. Most stroke-specific MBI studies to date have focused on psychological outcomes in small, selective samples, with limited attention to physiological effects [7].

Despite encouraging preliminary evidence, further rigorous and standardized research is needed, as few studies have employed head-to-head randomized designs that control for group dynamics. In this context, we conducted a co-designed feasibility trial comparing a yoga-based MBI for survivors’ post-stroke against an attention control program (lifestyle education and socialization), delivered via weekly 60-min sessions over 12 weeks. This trial was based on a small wait-list controlled trial, which established feasibility but was limited to survivors with hemiparesis [22]. Building on this prior research, we undertook a co-design process with survivors of stroke, carers, and clinicians to adapt and expand the program for broader applicability [23]. The resulting co-designed intervention was then evaluated in the current Phase II feasibility trial. The primary feasibility study outcomes of feasibility, acceptability, and broader exploratory outcomes will be reported separately. A similar sub-study exploring the impact on mental health and quality of life has been published elsewhere [24]. This present sub-study was exploratory and aimed to assess potential changes (magnitude and direction) in physiological risk factors. For the purposes of this sub-study, blood pressure and perceived stress were treated as primary exploratory outcomes, given their clinical relevance and high data completeness. Blood biomarkers (lipids and HbA1c) were considered secondary outcomes, as they were collected in a smaller subset of participants.

## 2. Materials and Methods

### 2.1. Trial Design and Ethics

This was a Phase II feasibility, prospective, parallel two-group, double-blinded, randomized controlled trial (RCT). The trial was approved by the Monash University Human Research Ethics Committee (MUHREC; ID: 1294) and conducted under the Declaration of Helsinki. The trial conforms to the ICMJE Recommendation for the Conduct, Reporting, Editing, and Publication of Scholarly Work in Medical Journals. Since our feasibility trial was conducted during COVID-19 pandemic, the CONSERVE-CONSORT checklist for RCTs, modified due to the COVID-19 Pandemic, and the CONSORT 2010 statement: extension to randomized pilot and feasibility trials were used to report the results (Supplementary Table S1) [25,26]. The trial was registered prospectively at the Australian New Zealand Clinical Trials Registry (ACTRN12620000105943) on 5 February 2020.

### 2.2. Participants

Participants were recruited from the Australian Stroke Clinical Registry (AuSCR) [27] if they had agreed to be contacted for research projects in response to their 90-day post-hospital admission follow-up survey. Recruitment was conducted between May 2021 and July 2023. Adult survivors of stroke were sent an invitation letter if discharged from the hospital within 3–18 months of a stroke, were living in the community within 30 km (km) of Monash University Brain Park facility (Clayton, VIC, Australia) and had agreed to be contacted for research studies. Participants were recruited from within a 30 km radius of the study site to minimize travel burden and ensure the feasibility of attendance.

### 2.3. Inclusion and Exclusion Criteria

Participants who returned an expression of interest were eligible and included if they: (1) had access to email and/or SMS (to access the home practice guide, and receive class reminders), (2) self-identified as users of electronic technology such as CD player (to access home practice guide), (3) had a baseline Modified Rankin Score of 0 (i.e., no disability) to 4 (i.e., may require some assistance but not constant care), (4) were able to give informed written consent and (5) understood two-step verbal commands. If they were already enrolled in another clinical trial, participation was allowed provided there was no risk of contamination between trials and minimal burden on the participant.

Participants were ineligible and excluded if: (1) undertaking a mindfulness program (meditation, Tai Chi, yoga) or had done these regularly (e.g., ≥monthly in frequency) in the 12 months before the stroke, (2) could not verbally communicate in English (due to constraints of the budget, translation of yoga intervention delivery by experts into other languages was not possible), (3) had significant language or communication impairments that would restrict the ability to participate, (4) poor prognosis (unlikely to survive to 12 weeks following randomization), (5) presence of contraindications to magnetic resonance imaging (MRI) (e.g., metallic or electronic implants not known to be safe), or (6) suffered from claustrophobia (i.e., MRI scanner is space constrained).

### 2.4. Recruitment and Informed Consent Process

Once eligible participants were identified in the AuSCR database, the recruitment procedure began with a two-phase mailout process based on a modified Dillman protocol to maximize the response rate [28]. Personalized letters, an explanatory statement, consent forms, an MRI eligibility screening checklist, and a pre-addressed postage-paid envelope were supplied to participants. Only participants and the statistician were blinded. In this feasibility trial, whereas the project coordinator (author TT) was not blinded. To maintain participant blinding, the trial was described in the explanatory statement in general terms as ‘a novel group-based class,’ without disclosing specific details of the intervention or attention control components. Interested potential participants provided their written consent by returning a signed form (i.e., informed written consent was obtained from all those participating). Once written consent was received, participants were contacted by the Project Coordinator by phone to confirm eligibility. Subsequently, baseline assessments were conducted at an agreed date and time at the Brain Park facility.

The COVID-19 pandemic significantly disrupted recruitment, with pauses from March 2020 to February 2021 and from July to September 2021 due to restrictions in Melbourne. Recruitment resumed only when conditions were safe. A risk mitigation plan was implemented, including room capacity limits and advising participants to report symptoms and avoid sessions if unwell. These measures were approved by MUHREC and the Brain Park facility manager.

### 2.5. Randomization

Once the baseline data were obtained, participants were randomly assigned to the intervention or the attention control arm. A 1:1 ratio randomization sequence, stratified by age and stroke severity, was conducted through the REDCap database system. An independent researcher created and uploaded the randomization sequence. Allocation was concealed using REDCap’s automated assignment function, ensuring that study staff enrolling participants could not predict or influence group assignment.

### 2.6. Intervention

The intervention (MBI) was adapted from an established yoga program [22], and refined through stakeholder consultation, including input from survivors of stroke, carers, allied healthcare professionals, and researchers. Details of the program have been reported elsewhere [23].

Participants attended a 12-week program comprising weekly 60-min group classes, delivered in a standardized format by trained yoga instructors, with up to five participants per class. Classes incorporated low-impact movement sequences, attention-focused breathing, and body awareness exercises, with individualized adaptations (e.g., chair support for seated or transitional poses) based on participant needs. Instructors met with participants in a preliminary session to address specific requirements, particularly for motor impairments, and to allay concerns about undertaking an MBI program.

Participants were also provided with audio-guided meditation sessions (20 min per session) for home practice at least three times per week, with access to recordings via a secure Google Drive folder. Home practice adherence was tracked using a weekly diary. Carers could attend classes if space allowed, provided their involvement did not disrupt group dynamics or the survivor’s participation. Expectations were clearly outlined and agreed upon prior to carer involvement.

This MBI was designed to accommodate individual needs while maintaining a standardized delivery, offering a comprehensive approach to promoting physical and psychological well-being in survivors of stroke.

### 2.7. Attention Control

The control group participated in a 12-week program designed to match the intervention group in duration, format, and level of social interaction. Each week, participants attended a 60-min “lifestyle education with socialization” session, semi-facilitated by two project staff. Sessions focused on general health and wellbeing topics identified through an unmet needs survey, such as nutrition, returning to work, and driving post-stroke. Mindfulness-related content or physical activity (e.g., meditation, yoga, Tai Chi) was deliberately excluded to avoid overlap with the intervention. Each session included a 15–20-min pre-recorded presentation by a content expert or a survivor of stroke/carer from partnering organizations, followed by an open discussion to promote peer engagement and social connection. The attention control was held at the same venue and scheduled for the same 12-week duration and weekly 60-min group format as the MBI, ensuring equivalence in contact time, setting, and social interaction.

### 2.8. Sub-Study Outcomes

Exploratory study outcomes were measured at two time points, i.e., baseline and post-intervention (13–15 weeks post-randomization), as described below for the results to be presented in this article.

### 2.9. Blood Pressure

Blood pressure was measured using the OMRON Automatic Blood Pressure Monitor (Ultra-Premium HEM-7320; Omron Healthcare Co., Ltd., Kyoto, Japan). Participants were asked to sit quietly and rest for 15 min with their legs uncrossed before measurement. Three measurements were taken from each participant at each assessment, with a three-minute rest between measurements. For analysis purposes, the mean of the second and third readings were used for the analysis.

### 2.10. Blood Lipids and Glycated Hemoglobin (HbA1c)

TC, HDL, and triglycerides (TGs) were determined using the CardioChek PA analyzer (point-of-care-system; PTS Diagnostics, Whitestown, IN, USA). LDL was calculated using the equation: LDL = TC – HDL − [TG/5]. Glycated hemoglobin reflects the proportion of glycated hemoglobin in the blood, indicating the average blood glucose level over the past 3 months. A finger-prick technique was used to collect capillary blood samples in those who consented to the test. Glycated hemoglobin was collected and analyzed using A1CNow+ Portable HbA1c Test Kit (point-of-care device; PTS Diagnostics, Whitestown, IN, USA). In this trial, a random reading was collected, i.e., participants were not required to fast before any measurements. All equipment was calibrated before data collection. Data on concomitant medications were not collected.

### 2.11. Stress

Stress was measured using both a biological marker (hair cortisol) and a self-report 10-item Perceived Stress Scale (PSS-10).

The hair samples (50–100 hairs) were cut from the posterior vertex of the participant’s scalp, as close to the scalp as possible, by a trained outcome assessor (SH). The hair samples were stored in an envelope with a mark indicating the root end and stored at room temperature in a non-airtight container until analysis and sent to the Hunter Medical Research Institute in batches. For the analysis of hair cortisol, the proximal 2 cm of the hair was cut, washed in isopropanol, and then left to dry. Hair was then ground into powder using Precelly’s machine, and 1.5 mL of methanol was added to extract cortisol. The hair samples were then incubated overnight on a shaker, centrifuged for 5 min at 10,000 rpm, and 1 mL of supernatant was collected and incubated to dry in order to evaporate the methanol. Finally, the hair samples were reconstituted in 100 µL of ELISA buffer and stored at −20 °C until needed. Cortisol was measured using an ELISA kit according to kit instructions (Stratech Scientific APAC Pty Ltd., Mona Vale, NSW, Australia) [29].

The PSS-10, a 10-item scale, was used to measure perceived psychological stress in all participants, asking them to rate how stressful they perceived their life to be during the previous month [30]. Item scores were rated on a 5-point scale (0 = never to 4 = very often), and the overall score ranges from 0 to 40, with higher scores suggesting higher levels of stress. The PSS-10 has been widely shown to have acceptable psychometric properties, ref. [30] and has been used previously in stroke survivors [31].

### 2.12. Sample Size

For this sub-study, included as part of our feasibility trial, we did not pre-specify a sample size. The feasibility trial aimed to recruit up to 30 participants per group. A sample size of 12 per group is often sufficient for feasibility studies to provide appropriate information [32]. The goal was not to estimate treatment effects with precision, but rather to examine preliminary trends to inform a future effectiveness trial.

### 2.13. Statistical Analysis

An intention-to-treat approach was used, meaning participants were analyzed according to the group to which they were initially randomized, irrespective of whether they completed the entire 12-week program. Data were checked for normality. We present overall summaries across both groups, such as mean and standard deviation or median (Q1, Q3), number and percentage, and proportion of missing data. To assess baseline differences between the control and intervention groups, chi-square tests were used for categorical variables, *t*-tests for continuous symmetric variables, and Wilcoxon rank-sum tests for skewed continuous variables. Baseline characteristics were compared between participants with and without blood sampling using independent-samples *t*-tests for continuous variables and chi-square tests for categorical variables to assess representativeness. As this sub-study of a phase II feasibility trial was not powered to detect statistically significant differences, exploratory analyses were conducted to examine the direction and magnitude of change between groups. Outcomes were analyzed for participants with complete baseline and follow-up data. Within-group differences were calculated and reported as effect sizes according to procedures described by Cohen: 0.2 to 0.50 = small to moderate; 0.51 to 0.80 = moderate to large; and >0.80 = large [33]. Between-group differences were explored using generalized linear mixed models that included program type and time (categorical) as fixed factors, with baseline scores entered as covariates and random intercepts to account for repeated measures [34]. Effect sizes are reported alongside model estimates to describe the magnitude of observed effects, consistent with the exploratory nature of this sub-study [24,35,36]. The significance level was set at *p* < 0.05 (two-tailed). All statistical analyses were done using Stata statistical software, version 18.0 (StataCorp LLC., College Station, TX, USA).

## 3. Results

**Figure 1:**
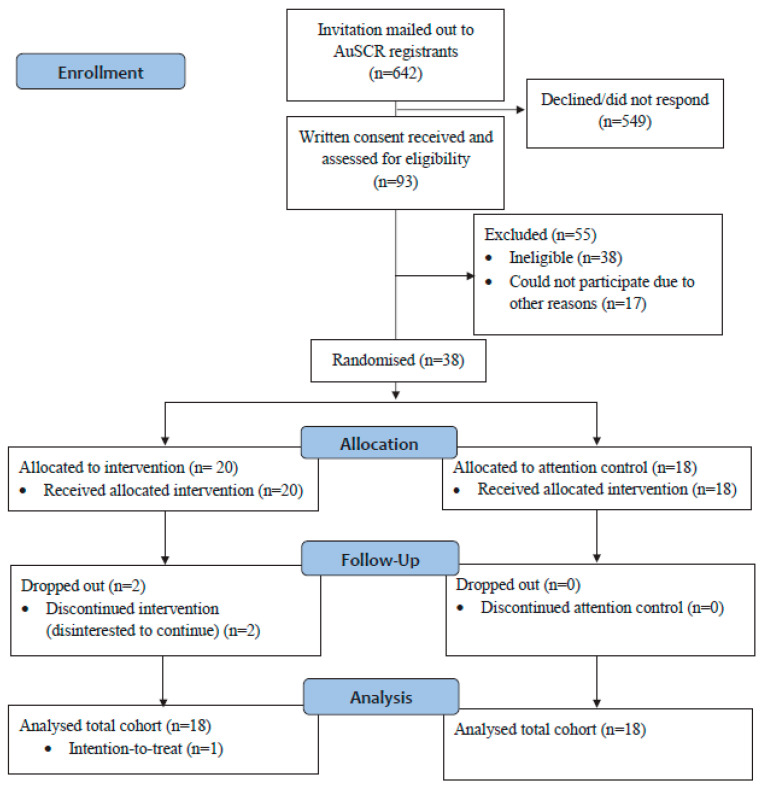
CONSORT flow diagram of participants in our feasibility trial.

Overall, 642 AuSCR registrants were invited to participate in our trial, of which 93 (median age 69, 65% male) provided consent for further eligibility assessment (Figure 1). Of these, 55 met the trial criteria, and 38 participants were recruited and randomized (Table 1). A total of 36 participants completed the trial, with a mean age of 69 years (72% male). Two dropped out from the intervention group (during the intervention) as they were disinterested to continue. Preliminary adherence data indicate that most participants (98%) attended at least 10 of the 12 sessions and engaged in prescribed home meditation at least three times per week. Full feasibility outcomes will be reported in a separate manuscript.

Within-group changes in physiological risk factors showed small to moderate, non-significant effect sizes (Table 2 and Table S1). Blood pressure effect sizes were larger in the MBI group compared to the attention control. Systolic blood pressure in the MBI group had a Cohen’s d Sof 0.34 compared to 0.10 in the control group, with mean systolic pressure decreasing from 136 mmHg at baseline to 131 mmHg post-intervention. Similarly, diastolic blood pressure in the MBI group showed a Cohen’s d of 0.41 versus 0.11 in the control group, with mean diastolic pressure decreasing from 82 mmHg at baseline to 78 mmHg post-intervention (Table 2).

Blood lipid analyses were available for only a very small subset of participants. Given this limitation, these data are presented in the Supplementary Materials (Table S1) as preliminary only. As lipid data were available for only a subset of participants, baseline characteristics were compared between those with and without blood sampling. No significant differences were found in age or gender (*p* > 0.05), indicating that the subsample with blood sampling was broadly representative of the full cohort. For participants with available data (n = 17), no notable changes were observed for HbA1c levels in either group (Table 2).

In terms of stress markers, there was a small improvement in perceived stress scores for the MBI group (effect size of 0.12) compared to no change in the control group (0.00), with a mean perceived stress score decreasing from 12 to 11. The MBI group showed a moderate reduction (Cohen’s d of 0.52) in hair cortisol, while the control group also experienced a smaller reduction (0.34), with a greater difference in mean hair cortisol (pg/mg) seen in the MBI group (Table 2).

To illustrate individual-level variability, we calculated the proportion of participants in the MBI group who demonstrated improvement from baseline to post-intervention. Specifically, 94% (17/18) showed a reduction in systolic blood pressure, 83% (15/18) reported lower perceived stress scores, and 86% (6/7) had reductions in hair cortisol levels. Although average effect sizes (Table 2) ranged from small to moderate (d = 0.12–0.52), these proportions indicate that most participants showed change in the expected direction, albeit with varying magnitude. No statistically significant between-group differences were observed for any of the measured outcomes using generalized linear modeling (Table 2).

## 4. Discussion

In this sub-study of a feasibility trial, we explored the effects of a novel MBI compared to a peer-based social group (attention control) on physiological biomarkers in community-dwelling survivors of strokes, an area that remains underinvestigated. The main findings were that, although between-group differences were not statistically significant, the MBI group showed preliminary trends toward improved blood pressure regulation and possible favorable changes in lipid profiles and physiological stress. Given the small sample size, these findings should be interpreted with caution but are valuable for informing outcome selection and sample size estimates for future adequately powered trials.

### 4.1. Blood Pressure

The larger effect sizes observed for systolic and diastolic blood pressure in the MBI group were not statistically significant but indicate preliminary trends toward improved blood pressure regulation in survivors of stroke, which may help reduce risks of recurrent strokes. Specifically, mean systolic pressure decreased by 5 mmHg and mean diastolic pressure decreased by 4 mmHg, both of which are recognized as clinically meaningful reductions [37]. Evidence from similar studies supports our findings, showing that MBIs, including practices like mindfulness-based stress reduction and paced breathing, can favorably moderate blood pressure. MBIs have been associated with reduced systolic blood pressure in cardiovascular and hypertensive populations, likely through the effects on the autonomic nervous system, which decrease sympathetic activation and promote relaxation [18,38]. Additionally, mindfulness with controlled and focused breathing has shown promise for short-term blood pressure reductions by stimulating the parasympathetic nervous system and moderating stress responses [12]. Research among people living with stroke suggests that MBIs can assist in managing the physical and emotional challenges associated with recovery, with noted improvements in mood and blood pressure regulation [16]. As high blood pressure is the most prevalent and modifiable risk factor for stroke, MBIs may offer a promising, non-pharmacological approach to suppose cardiovascular risk reduction and recovery, underscoring the need for larger, targeted trials.

### 4.2. Blood Lipids

Data on blood lipids were available for only a small subset of participants, particularly in the MBI group, therefore these analyses are presented in the Supplementary Materials for transparency. Within this limited sample, small but non-significant preliminary changes in LDL and HDL cholesterol are observed within the MBI group, while total cholesterol showing greater changes in the control group. No within-group differences were identified for the TC/HDL ratio, though TG levels showed divergent trends between groups. These exploratory findings are broadly consistent with prior studies suggesting that MBIs may support lipid regulation indirectly, through reductions in stress reactivity and improvements in adherence to health behaviors such as diet and physical activity [39,40,41]. Given the established role of LDL reduction in secondary stroke prevention and its central position in current clinical guidelines [4,42], even small improvements are of potential relevance. While HDL cholesterol plays a secondary role in risk stratification, favorable changes in both LDL and HDL have been associated with reduced risk of recurrent vascular events. However, due to funding constraints, point-of-care devices were only available for a subset of participants (groups 3 and 4), and blood samples were limited to these groups. Given the small sample size and use of point-of-care devices in only a subset of participants, these results should be interpretated cautiously. This limitation underscores the need for adequately powered studies with comprehensive biochemical sampling to better evaluate the potential role of MBIs in cardiovascular risk reduction among survivors of stroke [43,44].

### 4.3. Glycated Hemoglobin

No significant HbA1c changes were observed, however, given the small sample size, it remains uncertain whether MBIs influence glycemic control in this population. While previous researchers have indicated that MBIs, like yoga and mindfulness-based stress reduction, can lead to modest HbA1c reductions, the results are variable, often small, and not clinically relevant [45]. MBIs may effectively reduce stress and support emotional well-being, thereby indirectly benefiting metabolic health in those with chronic conditions like stroke [45,46]. Future research with larger samples is needed to clarify these potential indirect effects.

### 4.4. Stress

Reductions in both perceived stress and hair cortisol levels observed in this study suggest that MBIs may play a valuable role in post-stroke stress management. These findings align with previous research showing that mindfulness-based stress reduction and mindfulness-based cognitive therapy can significantly reduce stress, depression, and anxiety in vascular disease populations [18]. Yoga-based MBIs have been shown to modulate the autonomic nervous system and regulate the hypothalamic–pituitary–adrenal axis, reducing sympathetic activity, enhancing parasympathetic tone, and lowering cortisol levels, thereby improving physiological stress regulation [11]. The inclusion of hair cortisol as an objective measure of stress in our study offers a novel contribution. Unlike acute stress markers, hair cortisol reflects cumulative cortisol exposure over weeks to months and provides a stable non-invasive measure of long-term physiological stress. Its ease of collection, storage, and transport also makes it a practical tool for future clinical and research applications. Future studies should explore the mechanisms linking MBIs, stress physiology, and recovery outcomes, integrating both subjective and objective stress measures to strengthen the understanding of how these interventions may enhance post-stroke stress management. While promising trends were observed in blood pressure (clinically significant differences), lipid profiles, and stress markers within the MBI group, the lack of statistically significant between-group differences emphasizes the need for larger, adequately powered trials to validate these findings. These descriptive patterns also suggest the presence of potential responders within the MBI group and underscore the value of further investigation in a fully powered trial designed to explore individual treatment response.

### 4.5. Strengths and Limitations

A key strength of this sub-study is the inclusion within a robust head-to-head randomized trial design, directly comparing the MBI intervention to attention control, addressing the limitations of previous studies that relied on waitlist controls. By engaging both groups in real-time, this approach minimized biases associated with passive controls and ensured a more rigorous comparison. This design strengthens the evidence for the specific benefits of MBIs and sets a higher standard for future research.

A limitation of this substudy and feasibility trial is the small sample size, influenced by COVID-19-related recruitment challenges and restricted funding for point-of-care diagnostic devices, which reduced the capacity to detect definitive effects. COVID-19 restrictions primarily influenced recruitment, but we did not observe notable effects on retention or outcomes. A detailed analysis of recruitment feasibility will be reported separately. Small samples for some biomarkers (e.g., LDL, HDL, TG) further limit reliability and clinical interpretability, so these findings should be viewed as exploratory. While the point-of-care devices have demonstrated acceptable validity for rapid blood lipid and glucose measurements, these devices may be less precise than central laboratory methods. This limitation should be considered when interpreting the findings. Additionally, the absence of data on medications, particularly those impacting blood pressure and lipid levels, which are commonly prescribed to survivors of stroke, may have confounded the intervention’s effect on physiological outcomes. Medication data were not collected as the primary aim of this feasibility trial was to assess recruitment, retention, and acceptability. Future studies should incorporate detailed medication data to allow adjustment for their effects on physiological outcomes. Furthermore, in some cases, participants did not provide enough blood for the point-of-care device to function, and others did not have sufficient hair samples available for cortisol sampling, resulting in incomplete data. Restricting recruitment to within 30 km of the study site was intended to support recruitment, retention, and avoid loss to follow-up. We acknowledge that this criterion may have limited the generalizability of our cohort by excluding people able and willing to travel further than 30 km. 

As this was a small feasibility trial, observed within-group changes may have been influenced by random variation or regression to the mean rather than true intervention effects. These trends should therefore be considered preliminary indicators to guide the design and powering of future trials. While feasibility trials focus on real-world viability rather than statistical significance, the exploratory findings and limitations highlight the need for a full-scale RCT to assess clinical outcomes with adequate power and appropriate control of confounding variables.

## 5. Conclusions

Our findings suggest that MBIs may support secondary stroke prevention and cardiovascular health. While preliminary patterns in blood pressure, lipid profiles, and stress markers appeared favorable in the MBI group, the small sample size and exploratory design limit the ability to draw definitive conclusions. Importantly, this trial used a robust RCT design with a 1:1 active control, enhancing the reliability of observed trends compared to studies using waitlist controls. Future research should prioritize larger, adequately powered RCTs to evaluate MBIs as complementary therapies for reducing cardiovascular and metabolic risk in survivors of stroke.

## Figures and Tables

**Table 1:** Baseline characteristics of participants recruited to the trial.

Variables	Intervention N = 18 n (%)	Control N = 18 n (%)	*p*-Value
*Demographics*			
Age, median (Q1, Q3)	69 (64, 76)	73 (65, 78)	0.67
Female	5 (25)	5 (28)	0.85
*Stroke type*			
Ischemic	18 (90)	18 (100)	
Hemorrhagic	2 (10)	0 (0)	0.17
*Stroke Severity*			
NIHSS in hospital, median (Q1, Q3)	5 (3, 19)	4 (1, 14)	0.34
Able to walk during admission	10 (50)	9 (56) *	0.19
Slight to severe disability (mRS 2–4)	7 (37)	6 (35)	0.92

N = group total; n = count; Q1: Quartile 1; Q3: Quartile 3; NIHSS: National Institutes of Health Stroke Scale. * missing n = 2.

**Table 2:** Within and between-group differences in stress, blood pressure, glycated hemoglobin, and blood lipids in participants who completed the trial.

Outcome Measure	Within-Group Differences								Between-Groups Differences *	
		Attention Control				MBI				
	Sample Size (n)	Baseline Mean (SD)	Post-Intervention Mean (SD)	Cohen’s d *	Sample Size (n)	Baseline Mean (SD)	Post-Intervention Mean (SD)	Cohen’s d *	β Coefficient (95% CI)	*p*-Value
*Blood pressure*										
Systolic (mmHg)	18	140 (16.9)	139 (16.4)	0.10	18	136 (14.4)	131 (14.0)	0.34	−3.3 (−11.4, 4.8)	0.421
Diastolic (mmHg)	18	82 (8.4)	81 (7.6)	0.11	18	82 (8.2)	78 (11)	0.41	−3.2 (−8.7, 2.3)	0.249
*Stress*										
Perceived Stress Scale	18	14 (9.1)	14 (8.6)	0	18	12 (5.8)	11 (6.6)	0.12	−0.8 (−4.3, 2.8)	0.667
Hair cortisol (pg/mg)	7	15 (6.9)	13 (5.2)	0.34	8	15 (9.6)	11 (1.2)	0.52	−1.5 (−10.5, 7.5)	0.743
*Blood glucose*										
HBA1c (%)	7	6.3 (1.1))	6.5 (1.1)	−0.22	10	5.8 (0.6)	6.1 (0.8)	−0.30	0.2 (−0.2, 0.6)	0.393

MBI: Movement-based mindfulness intervention; SD: Standard deviation; CI: Confidence Interval; HBA1c: glycated hemoglobin. Cohen’s d: 0.2 to 0.50 = small to moderate effect size; 0.51 to 0.80 = moderate to large effect size; and >0.80 = large effect size [30]. * The models included program, time (treated as a categorical variable), and baseline scores as fixed covariates.

## Data Availability

The datasets used and analyzed in this study is available from the corresponding author on reasonable request. The data are not publicly available due to privacy or ethical restrictions.

## Supplementary Materials

The following supporting information can be downloaded at: https://www.mdpi.com/article/10.3390/healthcare13222940/s1; Table S1: Preliminary Analysis of Blood Lipid Outcomes Among Participants Who Completed the Trial.

## Glossary

AuSCR	The Australian Stroke Clinical Registry
BMI	Body mass index
HbA1C	Glycated hemoglobin
HDL	High-density lipoprotein
ICMJE	International Committee of Medical Journal Editors
LDL	Low-density lipoprotein
MBI	Mindfulness-based intervention
MUHREC	Monash University Human Research Ethics Committee
PSS	Perceived Stress Scale
RCT	Randomized controlled trial
SMS	Short message service
TC	Total cholesterol
TG	Triglyceride

## References

[1] Deloitte Access Economics (2013). The Economic Impact of Stroke in Australia.

[2] Flach C., Muruet W., Wolfe C.D.A., Bhalla A., Douiri A. (2020). Risk and Secondary Prevention of Stroke Recurrence: A Population-Base Cohort Study. Stroke.

[3] Hankey G.J. (2014). Secondary stroke prevention. Lancet Neurol..

[4] O’Donnell M.J., Chin S.L., Rangarajan S., Xavier D., Liu L., Zhang H., Rao-Melacini P., Zhang X., Pais P., Agapay S. (2016). Global and regional effects of potentially modifiable risk factors associated with acute stroke in 32 countries (INTERSTROKE): A case-control study. Lancet.

[5] Mohan K.M., Wolfe C.D.A., Rudd A.G., Heuschmann P.U., Kolominsky-Rabas P.L., Grieve A.P. (2011). Risk and Cumulative Risk of Stroke Recurrence. Stroke.

[6] Walter A.A., Van Puymbroeck M., Bosch P., Schmid A.A. (2022). Complementary and integrative health interventions in post-stroke rehabilitation: A systematic PRISMA review. Disabil. Rehabil..

[7] Thayabaranathan T., Immink M.A., Stevens P., Hillier S., Thrift A.G., Brodtmann A., Carey L., Kilkenny M.F., Cadilhac D.A. (2018). Understanding the potential for yoga and tai chi interventions to moderate risk factors for stroke—A scoping review. Future Neurol..

[8] Hagins M., States R., Selfe T., Innes K. (2013). Effectiveness of Yoga for Hypertension: Systematic Review and Meta-Analysis. Evid.-Based Complement. Altern. Med..

[9] McDermott K.A., Rao M.R., Nagarathna R., Murphy E.J., Burke A., Nagendra R.H., Hecht F.M. (2014). A yoga intervention for type 2 diabetes risk reduction: A pilot randomized controlled trial. BMC Complement. Altern. Med..

[10] Malinowski P. (2013). Neural mechanisms of attentional control in mindfulness meditation. Front. Neurosci..

[11] Pascoe M.C., Thompson D.R., Ski C.F. (2017). Yoga, mindfulness-based stress reduction and stress-related physiological measures: A meta-analysis. Psychoneuroendocrinology.

[12] Brenner J., LeBlang S., Lizotte-Waniewski M., Schmidt B., Espinosa P.S., DeMets D.L., Newberg A., Hennekens C.H. (2020). Mindfulness with paced breathing reduces blood pressure. Med. Hypotheses.

[13] Pal A., Srivastava N., Tiwari S., Verma N.S., Narain V.S., Agrawal G.G., Natu S.M., Kumar K. (2011). Effect of yogic practices on lipid profile and body fat composition in patients of coronary artery disease. Complement. Ther. Med..

[14] Manchanda S.C., Madan K. (2014). Yoga and meditation in cardiovascular disease. Clin. Res. Cardiol..

[15] Bhardwaj P., Kaur N., Malik N., Singh G., Pathania M., Anand A. (2024). Yoga and Mindfulness in the Prevention of Metabolic Diseases. Neuroscience of Yoga: Theory and Practice: Part II.

[16] Lawrence M., Booth J., Mercer S., Crawford E. (2013). A Systematic Review of the Benefits of Mindfulness-Based Interventions following Transient Ischemic Attack and Stroke. Int. J. Stroke.

[17] Thayabaranathan T., Andrew N.E., Immink M.A., Hillier S., Stevens P., Stolwyk R., Kilkenny M., Cadilhac D.A. (2017). Determining the potential benefits of yoga in chronic stroke care: A systematic review and meta-analysis. Top. Stroke Rehabil..

[18] Abbott R.A., Whear R., Rodgers L.R., Bethel A., Thompson Coon J., Kuyken W., Stein K., Dickens C. (2014). Effectiveness of mindfulness-based stress reduction and mindfulness based cognitive therapy in vascular disease: A systematic review and meta-analysis of randomised controlled trials. J. Psychosom. Res..

[19] Priya G., Kalra S. (2018). Mind-Body Interactions and Mindfulness Meditation in Diabetes. Eur. Endocrinol..

[20] Wang S., Zhang C., Sun M., Zhang D., Luo Y., Liang K., Xu T., Pan X., Zheng R., Shangguan F. (2023). Effectiveness of mindfulness training on pregnancy stress and the hypothalamic-pituitary-adrenal axis in women in China: A multicenter randomized controlled trial. Front. Psychol..

[21] Lee S.Y., Gathright E.C., Wu W.-C., Salmoirago-Blotcher E. (2023). Mindfulness-Based Interventions for Patients with Cardiovascular Disease: A Focused Review for Practicing Clinicians. Curr. Cardiol. Rep..

[22] Immink M.A., Hillier S., Petkov J. (2014). Randomised Controlled Trial of Yoga for Chronic Poststroke Hemiparesis: Motor Function, Mental Health, and Quality of Life Outcomes. Top. Stroke Rehabil..

[23] Thayabaranathan T., Immink M.A., Hillier S., Stolwyk R., Andrew N.E., Stevens P., Kilkenny M.F., Gee E., Carey L., Brodtmann A. (2021). Co-Designing a New Yoga-Based Mindfulness Intervention for Survivors of Stroke: A Formative Evaluation. Neurolology Int..

[24] Hancock S., Thayabaranathan T., Stolwyk R., Cameron J., Immink M.A., Hillier S., Kilkenny M.F., Brodtmann A., Carey L.M., Olaiya M. (2025). Anxiety, Depression, Fatigue, and Quality of Life Outcomes Following a Movement-Based Mindfulness or Social Group Program for Chronic Stroke: A Sub-Study of a Phase II Feasibility Randomised Controlled Trial. Mindfulness.

[25] Sandra M.E., Claire L.C., Michael J.C., Christine M.B., Sally H., Lehana T., Gillian A.L. (2016). CONSORT 2010 statement: Extension to randomised pilot and feasibility trials. BMJ.

[26] Orkin A.M., Gill P.J., Ghersi D., Campbell L., Sugarman J., Emsley R., Steg P.G., Weijer C., Simes J., Rombey T. (2021). Guidelines for Reporting Trial Protocols and Completed Trials Modified Due to the COVID-19 Pandemic and Other Extenuating Circumstances: The CONSERVE 2021 Statement. JAMA.

[27] Cadilhac D.A., Dalli L.L., Morrison J., Paice K., Carter H., Campbell B.C.V., Cloud G.C., Kilkenny M.F., Faux S., Hill K. (2023). The Australian Stroke Clinical Registry Annual Report 2022.

[28] Lannin N.A., Anderson C., Lim J., Paice K., Price C., Faux S., Levi C., Donnan G., Cadilhac D. (2013). Telephone follow-up was more expensive but more efficient than postal in a national stroke registry. J. Clin. Epidemiol..

[29] Gyawali P., Chow W.Z., Hinwood M., Kluge M., English C., Ong L.K., Nilsson M., Walker F.R. (2020). Opposing Associations of Stress and Resilience With Functional Outcomes in Stroke Survivors in the Chronic Phase of Stroke: A Cross-Sectional Study. Front. Neurol..

[30] Cohen S., Kamarck T., Mermelstein R. (1983). A global measure of perceived stress. J. Health Soc. Behav..

[31] Prather J.G., Stanfill A.G. (2023). An Integrative Review of the Utilization of the Perceived Stress Scale in Stroke Recovery. J. Neurosci. Nurs..

[32] Julious S.A. (2005). Sample size of 12 per group rule of thumb for a pilot study. Pharm. Stat..

[33] Cohen J. (1988). Statistical Power Analysis for the Behavioral Sciences.

[34] Detry M.A., Ma Y. (2016). Analyzing Repeated Measurements Using Mixed Models. JAMA.

[35] Lee E.C., Whitehead A.L., Jacques R.M., Julious S.A. (2014). The statistical interpretation of pilot trials: Should significance thresholds be reconsidered?. BMC Med. Res. Methodol..

[36] Lancaster G.A. (2015). Pilot and feasibility studies come of age!. Pilot Feasibility Stud..

[37] Kandzari D.E., Mahfoud F., Weber M.A., Townsend R., Parati G., Fisher N.D.L., Lobo M.D., Bloch M., Böhm M., Sharp A.S.P. (2022). Clinical Trial Design Principles and Outcomes Definitions for Device-Based Therapies for Hypertension: A Consensus Document From the Hypertension Academic Research Consortium. Circulation.

[38] Intarakamhang U., Macaskill A., Prasittichok P. (2020). Mindfulness interventions reduce blood pressure in patients with non-communicable diseases: A systematic review and meta-analysis. Heliyon.

[39] Amarenco P., Labreuche J. (2009). Lipid management in the prevention of stroke: Review and updated meta-analysis of statins for stroke prevention. Lancet Neurol..

[40] Loucks E.B., Britton W.B., Howe C.J., Eaton C.B., Buka S.L. (2015). Positive Associations of Dispositional Mindfulness with Cardiovascular Health: The New England Family Study. Int. J. Behav. Med..

[41] Ismond K.P., Bukutu C., Vohra S., Watson R.R., Zibadi S. (2018). Chapter 23—Mindfulness-Based Therapy and Heart Health. Lifestyle in Heart Health and Disease.

[42] Gentile C., Starnino L., Dupuis G., D’Antono B. (2022). Mindfulness-Based Stress Reduction in Older Adults at Risk for Coronary Artery Disease: A Pilot Randomized Trial. Clin. Gerontol..

[43] Chu P., Gotink R.A., Yeh G.Y., Goldie S.J., Hunink M.G. (2016). The effectiveness of yoga in modifying risk factors for cardiovascular disease and metabolic syndrome: A systematic review and meta-analysis of randomized controlled trials. Eur. J. Prev. Cardiol..

[44] Scott-Sheldon L.A.J., Gathright E.C., Donahue M.L., Balletto B., Feulner M.M., DeCosta J., Cruess D.G., Wing R.R., Carey M.P., Salmoirago-Blotcher E. (2020). Mindfulness-Based Interventions for Adults with Cardiovascular Disease: A Systematic Review and Meta-Analysis. Ann. Behav. Med..

[45] Sanogo F., Xu K., Cortessis V.K., Weigensberg M.J., Watanabe R.M. (2023). Mind- and Body-Based Interventions Improve Glycemic Control in Patients with Type 2 Diabetes: A Systematic Review and Meta-Analysis. J. Integr. Complement. Med..

[46] Love M.F., Sharrief A., Chaoul A., Savitz S., Beauchamp J.E.S. (2019). Mind-Body Interventions, Psychological Stressors, and Quality of Life in Stroke Survivors. Stroke.

