# Supplementary material for: Movement-Based Mindfulness vs. Attention Control for Modifying Physiological Risk in Chronic Stroke: Evidence from a Feasibility Trial
Source: Healthcare (Basel). 2025 Nov 17;13(22):2940. doi: 10.3390/healthcare13222940 (PMC12651954; doi:10.3390/healthcare13222940)
Supplement: Supplementary file 1 [file healthcare-13-02940-s001.zip › healthcare-3907259-supplementary.pdf]

Movement-Based Mindfulness vs. Attention Control for Modifying Physiological Risk in Chronic Stroke: Evidence from a Feasibility Trial

Tharshanah Thayabaranathan [1,2,3], Marina Paul [4,5,6], Frederick R, Walker [4,5,6], Shaun Hancock [1], Liam Allan [1,7], Maarten A. Immink [8], Susan Hillier [9], Monique F Kilkenny [1,10], Amy Brodtmann [2,11], Emma Gee [12], Leeanne M Carey [2,3,10,13], Rene Stolwyk [14], Julie Bernhard [2,10], Michael Nilsson [4,5,6] and Dominique A. Cadilhac [1,2,3,10]

1. School of Clinical Sciences at Monash Health, Monash University, Clayton, VIC 3168, Australia
2. Centre of Research Excellence in Stroke Rehabilitation, Heidelberg, VIC 3084, Australia
3. Centre of Research Excellence in Aphasia Recovery and Rehabilitation, Melbourne, VIC 3086, Australia
4. Heart and Stroke Research Program, Hunter Medical Research Institute, New Lambton Heights, NSW 2305, Australia
5. College of Health, Medicine and Wellbeing, the University of Newcastle, Newcastle, NSW 2308, Australia
6. Centre for Rehab Innovations, University of Newcastle, Callaghan, NSW 2308, Australia
7. Australian e-Health Research Centre, Commonwealth Scientific and Industrial Research Organization, Brisbane, QLD 4006, Australia
8. College of Nursing and Health Sciences, Flinders University, Adelaide, SA 5000, Australia
9. Innovation IMPlementation and Clinical Translation in Health (IIMPACT), Allied Health and Human Performance, University of South Australia, Adelaide, SA 5000, Australia
10. Stroke and Critical Care Theme, the Florey Institute of Neuroscience and Mental Health Heidelberg, Heidelberg, VIC 3084, Australia
11. School of Translational Medicine, Monash University, Melbourne, VIC 3168, Australia
12. Survivor of Stroke, Inspirational and Motivational Speaker, Cotham, VIC, 3101, Australia; emma@emma-gee.com
13. School of Allied Health, Human Service and Sport, LaTrobe University, Melbourne, VIC 3086, Australia
14. School of Psychological Sciences, Monash University, Clayton, VIC 3800, Australia

* Correspondence: tharshanah.thayabaranathan@monash.edu; Tel.: +613-7511-1968

Supplementary Materials

Table S1. Preliminary Analysis of Blood Lipid Outcomes Among Participants Who Completed the Trial

**Outcome measure**	**Within-group differences**								**Between-groups differences***	
	**Attention Control**				**MBI**					
	Sample size (n)	Baseline Mean (SD)	Post-intervention Mean (SD)	Cohen's d*	Sample size (n)	Baseline Mean (SD)	Post-intervention Mean (SD)	Cohen's d*	β Coefficient (95% CI)	*p*-value
Blood lipids										
HDL (mmol/L)	6	1.3 (0.3)	1.2 (0.2)	0.14	2	1.4 (0.3)	1.6 (0.2)	0.73	0.1 (-0.2, 0.3)	0.575
LDL (mmol/L)	3	1.4 (1.1)	1.4 (0.7)	0.02	2	1.4 (0.4)	1.0 (0.2)	1.28	-1.0 (-1.6, 0.5)	0.110
TG (mmol/L)	5	1.7 (1.0)	1.2 (0.4)	0.51	2	1.9 (1.0)	3.4(0.1)	-1.37	0.9 (-0.0, 1.8)	0.051
TC/HDL ratio	4	2.8 (1.3)	2.9 (0.8)	-0.07	2	2.8 (0.8)	3.0 (0.2)	-0.21	-0.7 (-1.6, 0.3)	0.157
TC (mmol/L)	4	3.6 (1.2)	3.3 (0.4)	0.30	2	3.7 (0.9)	3.6 (0.4)	0.15	-0.7 (-1.5, 0.1)	0.098

MBI: Movement-based mindfulness intervention; SD: Standard deviation; CI: Confidence Interval; HDL: High-density lipoprotein; LDL: Low-density lipoprotein; TG: Triglycerides; TC/HDL: Total cholesterol/high-density lipoprotein; TC: Total cholesterol; Cohen's d: 0.2 to 0.50 = small to moderate effect size; 0.51 to 0.80 = moderate to large effect size; and >0.80 = large effect size. * The models included program, time (treated as a categorical variable), and baseline scores as fixed covariates
